# A 10-point preoperative checklist: selecting patients for outpatient joint replacement surgery

**DOI:** 10.1186/s42836-024-00270-2

**Published:** 2024-09-12

**Authors:** Madhav Chowdhry, Edward J. McPherson

**Affiliations:** 1 Department of Continuing Education, University of Oxford, Oxford, OX1 2JA UK; 2 Department of Orthopaedic Surgery, David Geffen School of Medicine, UCLA, Los Angeles, CA 90404 USA

**Keywords:** Outpatient Surgery, Ambulatory Surgical Centre (ASC), Knee Replacement, Hip Replacement, Scoring Profile

## Abstract

**Background:**

With advancements in perioperative care, joint replacement (JR) surgery is undergoing a transition from opacified in-patient institutions to nimble out-patient Ambulatory Surgical Centers (ASC). The goal of JR in ASC setting is safe patient discharge with subsequent rehabilitation without readmission. Multi-modal preoperative rehabilitation (MMPR) is a novel field of perioperative care, encompassing comprehensive parameters to ensure smooth transition from fitness for surgery to JR in outpatient setting. At present, there are no open-access schemes for selecting patients qualified for JR in the ASC setting. In this article, we propose an evidence-based, 10-point systematic evaluation of patients with target endpoints for MMPR to qualify patients for JR as an outpatient procedure. This checklist is a non-proprietary scheme serving as an initial framework for surgeons exploring surgery in the ASC setting.

**Body:**

We introduce factors for a prehabilitation scheme, called Checklist Outpatient-Joint Replacement (CO-JR) to qualify patients for outpatient JR surgery. These factors have been developed based on an extensive literature review and the significant experience of authors to incorporate variables that drive a successful outpatient JR procedure. The factors include patient education, psychiatric & cognitive ability, medical fitness, musculoskeletal capability, financial ability, transportation access, patient motivation, information technology (IT) capabilities, along with ability to recover independently at home postoperatively. The CO-JR scheme is under the process of validation at multiple institutions. We introduce this as a starting point for collaborative development of an open-access scheme for all surgeons to learn and adapt as needed for their respective global region.

**Conclusion:**

We established a non-proprietary 10-point CO-JR scheme, serving as a framework for surgeons to successfully select patients for JR surgery in the ASC setting. We encourage concomitant validation of this scheme globally. Our goal is to reach an international consensus on an open-access scheme, available for all surgeons to enrol patients for JR in the ASC setting, but modifiable to accommodate regional needs.

## Background

Joint Replacement (JR) Surgery offers significant improvement in pain, functional outcomes and quality of life [1–3]. With the increasing volume of procedures performed globally, there is concern about rising healthcare costs [4–11]. Advancements in preoperative patient assessment and discharge logistics are allowing for a shift in JR procedures away from opacified in-patient institutions to nimble out-patient Ambulatory Surgical Centers (ASC) [12–16]. Multi-Modal Preoperative Rehabilitation (MMPR), also known as prehabilitation, is a novel field of perioperative care facilitating this transition.

Traditional preoperative care in JR involves education regarding surgical logistics and medical clearance. These discussions are generic and not patient-tailored. In contrast, MMPR encompasses comprehensive parameters to ensure that the patient will be fit to undergo JR in the outpatient setting. The goal of JR in the ASC setting is safe discharge and subsequent rehabilitation without readmission. MMPR scheme identifies parameters that require optimization. These include lifestyle modifications, optimization of medical comorbidities, patient education in the surgical process, psychological conditioning, physiotherapy and nutrition [17, 18]. The scheme is personalized for each patient to ensure a safe procedure and recovery in an ambulatory setting. It offers benefits to patients as well as the healthcare systems in efficiency of care. For patients, prehabilitation aims to improve postoperative functional outcomes and reduce the need for ancillary resources. For the healthcare system, prehabilitation offers a better delivery of value-based care by allowing for ambulatory discharge to home and completion of rehabilitation in an outpatient setting.

At present, there are no comprehensive, open-access schemes for selecting patients qualified for JR in the ASC setting. This article proposes a 10-point systematic evaluation of patients with target endpoints for MMPR to qualify patients for JR as an outpatient procedure. This checklist is a non-proprietary scheme, which we would like to be modifiable, to address differences in regional needs. Table 1 outlines our proposed Checklist Outpatient-Joint Replacement (CO-JR), qualifying patients for hip and knee replacement surgery in the ambulatory setting. The score range is from 0 to 10. To be qualified for outpatient JR surgery, we believe patients should have a score > 8, with a mandatory score of 1 (Yes) on Factors 1, 3, and 4. These factors were developed based on an extensive literature review and significant experience to incorporate all factors that drive a successful outpatient JR procedure. The factors include patient education, psychiatric and cognitive ability, medical fitness, musculoskeletal capability, financial ability, transportation access, patient motivation, information technology (IT) capabilities, along with ability to recover independently at home postoperatively (Tables 1 and 2).

**Table 1:** Proposed 10-point checklist for surgeons to enrol patients for joint replacement surgery in an outpatient setting

Checklist Outpatient-Joint Replacement (CO-JR)							
**No**	**Parameter**	**Optimal Value**	**Assessment Instruments**		**Score**		**Patient Remediation Plan** **(Evaluator to complete)**
						**Patient score**	
**1**	**Patient Education: Illness Coherence and Procedure Awareness**	Patient understands all aspects of JR Procedure, Recovery and his/her individual responsibility in this process Patient is evaluated for baseline psychological distress [19, 20], perception of illness (illness coherence) [21] and expectations of 24-h discharge following surgery [22–24]	Video tutorials or telemedicine class Final coordinator interview/1-on-1 session with patient		Yes = 1		
					No = 0		
**2**	**Patient Motivation**	Patient acknowledges the need for self-improvement and commitment to rehabilitation. The goal is to return to independent living [25–27] Patient articulates willingness to participate in Outpatient surgery protocol [23]	Reading a standardized script and assessing willingness to participate via a 10-point Modified Likert Scale (with 0 = unmotivated, unwilling and 10 = highly motivated, highly willing). A score of ≥ 5 would be sufficient to proceed with the surgery [26]		Yes = 1		
					No = 0		
**3**	**Medical Fitness, Habits, Nutrition**	Optimization of all medical comorbidities [28, 29] Normal albumin [30–33] and vitamin levels Negative Cotinine levels No Opiates/Tramadol within 4 months of surgery Initiate starting an anti-inflammatory diet [30, 34] Full capability to withstand surgical stress and postoperative anaemia & engage in rehabilitation (home and clinic)	Comprehensive Serum Laboratory testing and BMI $\le$ 32	WNL	Yes = 1 All six categories are fulfilled		
			Serum albumin [31–33]	≥ 3.5 g/dL			
			HbA1C levels	≤ 7%			
			Serum Cotinine [35, 36]	< 8 ng/dL	No = 0		
			STOP-Bang Index (Sleep Apnoea survey) [37]	0–2			
			UCLA Patient Medical Risk Stratification	< 3			
**4**	**Physical Musculoskeletal Capability**	Maximize preoperative strength, balance, agility **without** causing debilitating pain and joint inflammation Minimising postoperative fall risk Preoperative pain and inflammation must be kept at a minimum to avoid postoperative edema and excess pain cycle	6 Minute Walk Test (with/without aid) [38]	Yes	Yes = 1 Both categories were fulfilled		
			CMT (Table 2): $\ge$ 7 sit-to-stand repetitions in 30 s	Yes	No = 0		
**5**	**Computer & Internet Capability**	Able to engage in Telemedicine Visits Independently	Interview & in-home assessment for telemedicine		Yes (able) = 1		
					No (Not able) = 0		
**6**	**Perioperative In-Home Support**	Designated, on-demand, 24 × 7 (home-aid/family member) support available for 8 weeks post-op	Interview with patient, family & PCP to ensure such availability		Yes (arranged) = 1		
					No (Not arranged) = 0		
**7**	**Transportation**	On-demand access in a timely fashion for scheduled and unscheduled healthcare visits	Interview with patient & preoperative transportation status evaluation		Yes (available) = 1		
					No (Not available) = 0		
**8**	**Financial Ability**	Ability to cover all expected and unexpected charges related to joint recovery Ability to pre-pay down payment for surgical procedure	Occupational “time off” benefits (to include designated family helper) Interview with a financial coordinator		Yes (Can cover) = 1		
					No (Cannot cover) = 0		
**9**	**Postoperative Self Sufficiency**	Patient is self-sufficient and willing to engage during the recovery phase	Interview with patient, family & PCP In-home assessment for food stores and hygiene		Yes (Capable) = 1		
					No (Not capable) = 0		
**10**	**Perioperative Cognitive Ability**	Low likelihood to become confused and/or disoriented after going home from surgery No history of postoperative delirium No dementia & fully engaged in preoperative evaluation process	CAM (Confusion Assessment Method) [39] by Nurse navigator	< 2	Yes (Pass) = 1		
					No (Fail) = 0		

1. *PCP* Primary Care Physician, *WNL* Within Normal Limits, *CMT* Chowdhry McPherson Test (In review), *ADL* Activity of Daily Living

MMPR Rules for CO-JR:

✓ Items 1, 3 & 4 must be = 1 (Yes); Score > 8 required to qualify for JR in ASC

**Table 2:** Chowdhry-McPherson Test (CMT) for preoperative musculoskeletal capability assessment in joint replacement surgery

Chowdhry McPherson Test (CMT)^a^ explanation
**Rationale:** 1. The patient must be able to get out of bed, onto & out of the toilet, and into & out of the couch at home without becoming encumbered
**Test Description:** 1. Patient sits on a chair with knees at 90 degrees, feet flat on the floor. The chair should have no side arms 2. The patient is asked to perform repetitive sit-to-stand as many times as possible in 30 s. The patient is timed by the examiner 3. A passing grade is $\ge$ 7 repetitions/30 s

^a^Testing is in validation at the present time

One specific area in MMPR that we wish to emphasize is preoperative musculoskeletal training, which is in debate. The assumption is that preoperative musculoskeletal training will confer better postoperative recovery. Training methods include muscle resistance exercises, joint flexibility and step training [40]. The duration as well as frequency varies. However, in recent North American hip and knee national meetings, experts raised concerns regarding overzealous preoperative musculoskeletal training, which can cause polyarticular joint inflammation and excess musculoskeletal pain. Preoperative edema and pain adversely affect postoperative recovery. Hence, we recommend emphasis on keeping the joint “calm” and focusing on balance and gait training.

We acknowledge the 10-point CO-JR scoring system is yet to be validated in clinical trials. However, we introduced this as a starting point for the collaborative development of an open-access scheme for all surgeons to learn and adapt as needed for their respective global regions. Our future goal is to validate the scheme locally, but we encourage concomitant validation at other global centers. The goal is to continuously modify this scheme to maximize the outcomes for the ASC setting. We encourage input from all disciplines that interact with patient care in the ASC setting (nursing care, counsellors, physical/occupational therapists, psychiatrists/psychologists, primary care physicians, and orthopaedists).

## Conclusion

Our goal is to develop a non-proprietary, open access 10-point CO-JR scheme, developed by the collaborative effort of surgeons across the world, serving as a framework for successfully selecting patients for JR surgery in the ASC setting. We acknowledge that the needs of global populations vary, and the available medical resources are not alike. In the future, we envision a Modified CO-JR for various countries requiring different needs suiting their local ethnic and demographic variances, e.g., Modified CO-JR India, Modified CO-JR Nigeria, Modified CO-JR New Zealand, Modified CO-JR USA etc. The proposed scheme is aimed to serve as a benchmark and is currently under the process of validation. With this initial proposal, we encourage concomitant input and validation to create a common, global platform for JR in the ASC setting. In the future, we would encourage an in-person consensus meeting to further expand this grading system.

## Data Availability

There was no associated data with the perspective manuscript.

## References

[1] Neuprez A, et al. Total joint replacement improves pain, functional quality of life, and health utilities in patients with late-stage knee and hip osteoarthritis for up to 5 years. Clin Rheumatol. 2020;39(3):861–71. 10.1007/s10067-019-04811-y. 31720892 10.1007/s10067-019-04811-y

[2] Dailiana ZH, et al. Patient-reported quality of life after primary major joint arthroplasty: a prospective comparison of hip and knee arthroplasty. BMC Musculoskelet Disord. 2015;16:366. 10.1186/s12891-015-0814-9. 26612135 10.1186/s12891-015-0814-9 PMC4660648

[3] Berger RA. A comprehensive approach to outpatient total hip arthroplasty. Am J Orthop (Belle Mead NJ). 2007;36(9 Suppl):4–5. [Online]. Available: https://www.ncbi.nlm.nih.gov/pubmed/17948159. 17948159

[4] Inacio MCS, Paxton EW, Graves SE, Namba RS, Nemes S. Projected increase in total knee arthroplasty in the United States - an alternative projection model. Osteoarthr Cartil. 2017;25(11):1797–803. 10.1016/j.joca.2017.07.022. 10.1016/j.joca.2017.07.022 28801208

[5] Losina E, et al. Cost-effectiveness of total knee arthroplasty in the United States: patient risk and hospital volume. Arch Intern Med. 2009;169(12):1113–21. 10.1001/archinternmed.2009.136. discussion 1121–2, Jun 22 2009. 19546411 10.1001/archinternmed.2009.136 PMC2731300

[6] Inacio MCS, Graves SE, Pratt NL, Roughead EE, Nemes S. Increase in total joint arthroplasty projected from 2014 to 2046 in Australia: a conservative local model with international implications. Clin Orthop Relat Res. 2017;475(8):2130–7. 10.1007/s11999-017-5377-7. 28488253 10.1007/s11999-017-5377-7 PMC5498389

[7] Culliford D, et al. Future projections of total hip and knee arthroplasty in the UK: results from the UK clinical practice research datalink. Osteoarthr Cartil. 2015;23(4):594–600. 10.1016/j.joca.2014.12.022. 10.1016/j.joca.2014.12.022 25579802

[8] Bumpass DB, Nunley RM. Assessing the value of a total joint replacement. Curr Rev Musculoskelet Med. 2012;5(4):274–82. 10.1007/s12178-012-9139-6. 23054621 10.1007/s12178-012-9139-6 PMC3702749

[9] Aynardi M, Post Z, Ong A, Orozco F, Sukin DC. Outpatient surgery as a means of cost reduction in total hip arthroplasty: a case-control study. HSS J. 2014;10(3):252–5. 10.1007/s11420-014-9401-0. 25264442 10.1007/s11420-014-9401-0 PMC4171452

[10] Eom SH, Bamne AB, Chowdhry M, Chae IS, Kim TK. Bibliometric analysis of orthopedic literature on total knee arthroplasty in asian countries: a 10-year analysis. Knee Surg Relat Res. 2015;27(3):149–55. 10.5792/ksrr.2015.27.3.149. 26389067 10.5792/ksrr.2015.27.3.149 PMC4570949

[11] Machhindra MV, Kang JY, Kang YG, Chowdhry M, Kim TK. Functional outcomes of a new mobile-bearing ultra-congruent TKA system: comparison with the posterior stabilized system. J Arthroplasty. 2015;30(12):2137–42. 10.1016/j.arth.2015.06.011. 26187388 10.1016/j.arth.2015.06.011

[12] Berger RA, Jacobs JJ, Meneghini RM, Della Valle C, Paprosky W, Rosenberg AG. Rapid rehabilitation and recovery with minimally invasive total hip arthroplasty. Clin Orthop Relat Res. 2004(429):239–47. 10.1097/01.blo.0000150127.80647.80. 10.1097/01.blo.0000150127.80647.80 15577494

[13] Berger RA, Sanders S, Gerlinger T, Della Valle C, Jacobs JJ, Rosenberg AG. Outpatient total knee arthroplasty with a minimally invasive technique. J Arthroplasty. 2005;20(7 Suppl 3):33–8. 10.1016/j.arth.2005.05.021. 16214000 10.1016/j.arth.2005.05.021

[14] Bertin KC. Minimally invasive outpatient total hip arthroplasty: a financial analysis. Clin Orthop Relat Res. 2005;435:154–63. 10.1097/01.blo.0000157173.22995.cf. 10.1097/01.blo.0000157173.22995.cf 15930933

[15] Kolisek FR, McGrath MS, Jessup NM, Monesmith EA, Mont MA. Comparison of outpatient versus inpatient total knee arthroplasty. Clin Orthop Relat Res. 2009;467(6):1438–42. 10.1007/s11999-009-0730-0. 19224306 10.1007/s11999-009-0730-0 PMC2674170

[16] Chowdhry M, Bamne AB, Na YG, Kang YG, Kim TK. Prevalence and predictors of post-operative coronal alignment outliers and their association with the functional outcomes in navigated total knee arthroplasty. J Arthroplasty. 2014;29(12):2357–62. 10.1016/j.arth.2014.07.015. 25113784 10.1016/j.arth.2014.07.015

[17] Durrand J, Singh SJ, Danjoux G. Prehabilitation. Clin Med (Lond). 2019;19(6):458–64. 10.7861/clinmed.2019-0257. 31732585 10.7861/clinmed.2019-0257 PMC6899232

[18] Hughes MJ, Hackney RJ, Lamb PJ, Wigmore SJ, Christopher Deans DA, Skipworth RJE. Prehabilitation before major abdominal surgery: a systematic review and meta-analysis. World J Surg. 2019;43(7):1661–8. 10.1007/s00268-019-04950-y. 30788536 10.1007/s00268-019-04950-y

[19] Bay S, Kuster L, McLean N, Byrnes M, Kuster MS. A systematic review of psychological interventions in total hip and knee arthroplasty. BMC Musculoskelet Disord. 2018;19(1):201. 10.1186/s12891-018-2121-8. 30037341 10.1186/s12891-018-2121-8 PMC6055334

[20] Kooner S, et al. Do psychiatric disorders affect patient reported outcomes and clinical outcomes post total hip and knee arthroplasty? SAGE Open Med. 2021;9:20503121211012256. 10.1177/20503121211012254. 33996082 10.1177/20503121211012254 PMC8107666

[21] Levinger P, et al. Development and validation of a questionnaire assessing discrepancy between patients’ pre-surgery expectations and abilities and post-surgical outcomes following knee replacement surgery. Knee Surg Sports Traumatol Arthrosc. 2016;24(10):3359–68. 10.1007/s00167-014-3432-4. 25423872 10.1007/s00167-014-3432-4

[22] Husain A, Lee GC. Establishing realistic patient expectations following total knee arthroplasty. J Am Acad Orthop Surg. 2015;23(12):707–13. 10.5435/JAAOS-D-14-00049. 26493969 10.5435/JAAOS-D-14-00049

[23] Filbay SR, Judge A, Delmestri A, Arden NK, Group COS. Evaluating patients’ expectations from a novel patient-centered perspective predicts knee arthroplasty outcome. J Arthroplasty. 2018;33(7):2146-2152 e4. 10.1016/j.arth.2018.02.026. 29544972 10.1016/j.arth.2018.02.026 PMC6005343

[24] Conner-Spady BL, Bohm E, Loucks L, Dunbar MJ, Marshall DA, Noseworthy TW. Patient expectations and satisfaction 6 and 12 months following total hip and knee replacement. Qual Life Res. 2020;29(3):705–19. 10.1007/s11136-019-02359-7. 31741216 10.1007/s11136-019-02359-7

[25] Frankel L, et al. Osteoarthritis patients’ perceptions of “appropriateness” for total joint replacement surgery. Osteoarthritis Cartilage. 2012;20(9):967–73. 10.1016/j.joca.2012.05.008. 22659599 10.1016/j.joca.2012.05.008

[26] Hawker GA, Wright JG, Badley EM, Coyte PC. Perceptions of, and willingness to consider, total joint arthroplasty in a population-based cohort of individuals with disabling hip and knee arthritis. Arthritis Rheum. 2004;51(4):635–41. 10.1002/art.20524. 15334438 10.1002/art.20524

[27] Harris IA, Harris AM, Naylor JM, Adie S, Mittal R, Dao AT. Discordance between patient and surgeon satisfaction after total joint arthroplasty. J Arthroplasty. 2013;28(5):722–7. 10.1016/j.arth.2012.07.044. 23462496 10.1016/j.arth.2012.07.044

[28] Krueger CA, Kozaily E, Gouda Z, Chisari E, Courtney PM, Austin MS. Canceled total joint arthroplasty: who, what, when, and why? J Arthroplasty. 2021;36(3):857–62. 10.1016/j.arth.2020.09.006. 33032875 10.1016/j.arth.2020.09.006

[29] Seward MW, Chen AF. Obesity, preoperative weight loss, and telemedicine before total joint arthroplasty: a review. Arthroplasty. 2022;4(1):2. 10.1186/s42836-021-00102-7. 35005434 10.1186/s42836-021-00102-7 PMC8723914

[30] Schroer WC, LeMarr AR, Mills K, Childress AL, Morton DJ, Reedy ME. 2019 Chitranjan S. Ranawat Award: Elective joint arthroplasty outcomes improve in malnourished patients with nutritional intervention: a prospective population analysis demonstrates a modifiable risk factor. Bone Joint J. 2019;101-B(7_Supple_C):17–21. 10.1302/0301-620X.101B7.BJJ-2018-1510.R1. 31256648 10.1302/0301-620X.101B7.BJJ-2018-1510.R1

[31] Eminovic S, et al. Malnutrition as predictor of poor outcome after total hip arthroplasty. Int Orthop. 2021;45(1):51–6. 10.1007/s00264-020-04892-4. 33244636 10.1007/s00264-020-04892-4 PMC7801298

[32] Rudasill SE, Ng A, Kamath AF. Preoperative serum albumin levels predict treatment cost in total hip and knee arthroplasty. Clin Orthop Surg. 2018;10(4):398–406. 10.4055/cios.2018.10.4.398. 30505406 10.4055/cios.2018.10.4.398 PMC6250962

[33] Fryhofer GW, Sloan M, Sheth NP. Hypoalbuminemia remains an independent predictor of complications following total joint arthroplasty. J Orthop. 2019;16(6):552–8. 10.1016/j.jor.2019.04.019. 31660022 10.1016/j.jor.2019.04.019 PMC6806651

[34] Gillis C, Carli F. Promoting perioperative metabolic and nutritional care. Anesthesiology. 2015;123(6):1455–72. 10.1097/ALN.0000000000000795. 26248016 10.1097/ALN.0000000000000795

[35] Moyer TP, et al. Simultaneous analysis of nicotine, nicotine metabolites, and tobacco alkaloids in serum or urine by tandem mass spectrometry, with clinically relevant metabolic profiles. Clin Chem. 2002;48(9):1460–71. [Online]. Available: https://www.ncbi.nlm.nih.gov/pubmed/12194923. 12194923 10.1093/clinchem/48.9.1460

[36] Hart A, Rainer WG, Taunton MJ, Mabry TM, Berry DJ, Abdel MP. Cotinine testing improves smoking cessation before total joint arthroplasty. J Arthroplasty. 2019;34(7S):S148–51. 10.1016/j.arth.2018.11.039. 30579712 10.1016/j.arth.2018.11.039

[37] Chung F, Abdullah HR, Liao P. STOP-Bang questionnaire: a practical approach to screen for obstructive sleep apnea. Chest. 2016;149(3):631–8. 10.1378/chest.15-0903. 26378880 10.1378/chest.15-0903

[38] Ko V, Naylor JM, Harris IA, Crosbie J, Yeo AE. The six-minute walk test is an excellent predictor of functional ambulation after total knee arthroplasty. BMC Musculoskelet Disord. 2013;14:145. 10.1186/1471-2474-14-145. 23617377 10.1186/1471-2474-14-145 PMC3644243

[39] Dworkin A, Lee DS, An AR, Goodlin SJ. A simple tool to predict development of delirium after elective surgery. J Am Geriatr Soc. 2016;64(11):e149–53. 10.1111/jgs.14428. 27650453 10.1111/jgs.14428

[40] Topp R, Swank AM, Quesada PM, Nyland J, Malkani A. The effect of prehabilitation exercise on strength and functioning after total knee arthroplasty. PM R. 2009;1(8):729–35. 10.1016/j.pmrj.2009.06.003. 19695525 10.1016/j.pmrj.2009.06.003

